# Domino Liver Transplantation: Anesthetic Considerations for a Patient With Maple Syrup Urine Disease and Type I Diabetes Mellitus

**DOI:** 10.7759/cureus.67983

**Published:** 2024-08-27

**Authors:** Colton Southard-Goebel, Ricardo A Serrano

**Affiliations:** 1 Department of Anesthesiology, Indiana University School of Medicine, Indianapolis, USA; 2 Department of Anesthesiology, University of North Carolina at Chapel Hill, Chapel Hill, USA

**Keywords:** type 1 diabetes mellitus, liver transplant anesthesia, maple syrup urine disease (msud), perioperative management, domino liver transplant

## Abstract

In this report, we describe the case of a patient with concomitant maple syrup urine disease (MSUD) and type I diabetes mellitus (T1DM) who underwent domino liver transplantation (DLT) , and the associated perioperative management. To the best of our knowledge, a DLT in an adult with both MSUD and T1DM has not been previously reported in the literature. Intensive care admission with multidisciplinary oversight is necessary for metabolic preconditioning prior to surgery. The complex interplay between these two disease processes presented with grossly elevated baseline insulin requirements and refractory intraoperative hyperglycemia. Following the successful procedure, the patient maintained excellent glycemic control on a normal diet. Four months post transplant, the patient presented with mild to moderate cellular graft rejection.

## Introduction

Maple syrup urine disease (MSUD) is an autosomal recessive, inborn error of metabolism, resulting in variable dysfunction of branched-chain ɑ-ketoacid dehydrogenase (BCKAD) [1]. This enzyme mediates the catabolism of branched-chain amino acids (BCAAs) such as valine, leucine, and isoleucine. Most BCAA metabolism occurs in skeletal muscle, with 10-15% occurring in the liver and kidney. If left untreated, the accumulation of toxic BCAA metabolites can result in irreversible neurological damage. First-line management of MSUD involves dietary restriction of BCAAs, essential amino acid supplementation, and avoidance of catabolic states, all accomplished alongside rigorous metabolic monitoring [1]. This life-long diet and lifestyle are physically, emotionally, and financially taxing for patients and their families [2]. Notwithstanding optimal treatment, crises may occur during inevitable infection, fasting, and stress, in some cases even leading to death [3]. Some data suggest a decline in intellectual ability and intelligence quotient (IQ) scores, even with strict adherence to recommended dietary measures [4].

Liver transplantation is an internationally recognized treatment for MSUD [2]. It is possible to transplant the liver of patients with MSUD, who have normal synthetic and metabolic liver function, to patients who do not have MSUD [5]. Domino (or sequential) liver transplantation (DLT) involves using the explanted liver of one liver transplant recipient as the graft for another patient. Complex, technically difficult, and time-consuming hepatectomy can be expected due to the need to preserve the native/explanted liver for a second recipient [6]. DLT is viable in MSUD due to a minimal (~15%) metabolic deficit in BCAA metabolism for the MSUD graft recipient, given normal extrahepatic enzyme activity, with no risk of MSUD development [2,5,6]. Provision of another, often cadaveric, liver graft for the MSUD patient is curative, ensuring sufficient BCAA metabolism to follow a normal diet without the risk of neurological sequelae [2].

In the current literature, there exists one case report documenting the co-occurrence of both MSUD and type I diabetes mellitus (T1DM) [7]. A DLT case with both MSUD and T1DM in an adult has not been previously reported as per our literature search. In this report, we describe the case of a patient with concomitant MSUD and TIDM who underwent DLT and the associated perioperative management. The graft was obtained from a donor with brain death at an external center.

A written Health Insurance Portability and Accountability Act (HIPAA) authorization to disclose existing protected health information was obtained for this case report. This manuscript adheres to the Anesthesia Case Report (ACRE) checklist [8].

## Case presentation

A 29-year-old male with a body mass index of 26.9 kg/m^2^ and a past medical history of MSUD, insulin-dependent T1DM, and gastroesophageal reflux disease presented for DLT. The patient had normal cognition at baseline and was diagnosed with MSUD pre-symptomatically at birth and with T1DM at 12 years of age. He was heterozygous for an in-frame deletion of Phe263del (F263del) in the *BCKDHA* gene, with conflicting interpretations of pathogenicity. His disease course had been complicated by frequent hospitalizations for leucine encephalopathic crises, with and without concomitant diabetic ketoacidosis (DKA), the most recent of which occurred less than eight months prior to admission to the intensive care unit. These exacerbations increased in frequency and were historically difficult to manage due to the interplay between MSUD and diabetes. His MSUD crises were typically associated with visual and auditory hallucinations, confusion, and occasionally nausea and vomiting.

One month before the procedure, he was granted an exception score and was listed for liver transplantation. The patient was admitted 10 days before the procedure for tentative liver transplantation, which was canceled due to an unsuitable donor graft. He removed his insulin pump 30-45 minutes prior to admission for his tentative procedure with an asymptomatic blood glucose of 380 mg/dL. After appropriate medical management, he was discharged.

Ten days later, the patient was admitted to the surgical intensive care unit (SICU) for metabolic optimization with medical management by transplant, endocrine, genetics, nutrition, and SICU teams. Pretransplant cardiopulmonary testing demonstrated a mild interatrial shunt and an otherwise unremarkable echocardiogram. Additionally, a cardiac computed tomography (CT) angiography showed no evidence of coronary artery disease. Other imaging studies included an abdominal CT with no variant hepatic vasculature and an abdominal MRI with a morphologically healthy liver.

On admission, he was stable and asymptomatic. The preoperative electrocardiogram demonstrated normal sinus rhythm. Preoperative laboratory data is recorded in Table 1. At home, the patient was on insulin tandem pump therapy with continuous glucose monitoring and a basal rate of 1.75-2 units/hour depending on physical activity. Pump boluses were administered with carbohydrate intake.

**Table 1:** Preoperative laboratory values ^*^Using minimum of 1 mg/dL; ^†^Using maximum of 137 mmol/L; ^‡^Using maximum of 3.5 g/dL HbA1c: glycated hemoglobin; MELD: Model for End-Stage Liver Disease; Na: sodium; INR: international normalized ratio

Laboratory tests	Value	Reference range
HbA1c	8.30%	<6.5%
MELD 3.0 score	7	6-40
MELD-sodium	7	6-40
Serum Creatinine	0.74 mg/dL^*^	0.6-1.2 mg/dL
Serum Sodium	138 mmol/L^†^	135-145 mmol/L
Total Bilirubin	1.2 mg/dL	0.0-1.0 mg/dL
Serum Albumin	3.8 g/dL^‡^	3.5-5.0 g/dL
INR	1.04	< 1.1

On the day of surgery, his calculated Model for End-Stage Liver Disease (MELD)-sodium (MELD-Na) and MELD 3.0 score was 7. Until the NPO (nil per os; nothing by mouth) was made, the patient continued his home insulin management and low-protein diet, including four daily packets of MSUD Express™ 20 divided over three servings with sugar-free beverages and food. Other intact protein intake was limited to a maximum of 20 g per day from vegetables, fruits, and starches. The NPO status was initiated 12 hours preoperatively and central venous access via the left internal jugular vein was obtained prior to initiation of custom low-protein total parenteral nutrition (TPN) per endocrinology to avoid a catabolic response perioperatively. The TPN was formulated with amino acids in the form of Clinisol SF 15% 10g, dextrose content of 350 g (with a relative glucose infusion rate of 3 mg/kg/minute) in normal saline without potassium. The insulin pump was discontinued, and intravenous insulin at a dose of 6.5 units/hour was initiated. In the event of TPN depletion or expiration during surgery, highly concentrated dextrose (35% in normal saline) was to be used as a second-line therapy. EndoTool® (Monarch Medical Technologies, Charlotte, North Carolina, United States) glucose monitoring was initiated in hyperglycemic mode with standard settings with a target range of 110-140 mg/dL, compared to standard 140-180 mg/dL.

In the preoperative physical exam, his vital signs, airway, and the rest of the physical exam were within normal limits. The patient received pre-medication with midazolam and was induced with fentanyl, lidocaine, propofol, and rocuronium. He received 500 mg of methylprednisolone and tacrolimus for immunosuppression with a blood trough goal of 8-10 ng/mL. Piperacillin-tazobactam was administered every two hours intraoperatively per standard dosing. His hemodynamic monitoring included standard American Society of Anesthesiologists (ASA) monitors, an arterial line, a 9FR triple-lumen catheter, and transesophageal echocardiography. Point-of-care glucose checks were conducted hourly per standard protocol.

**Figure 1:**
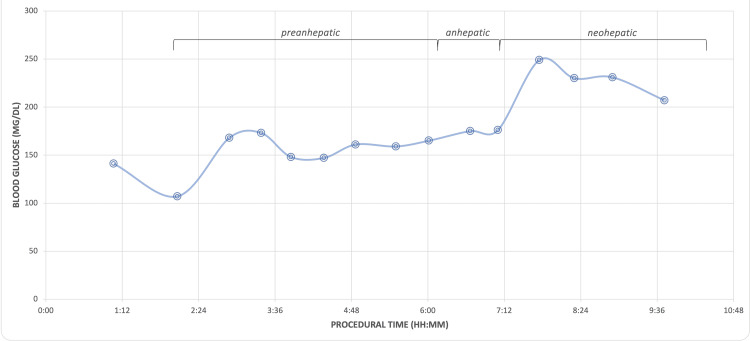
Intraoperative blood glucose concentration during domino liver transplantation Image Credit: Colton Southard-Goebel

**Figure 2:**
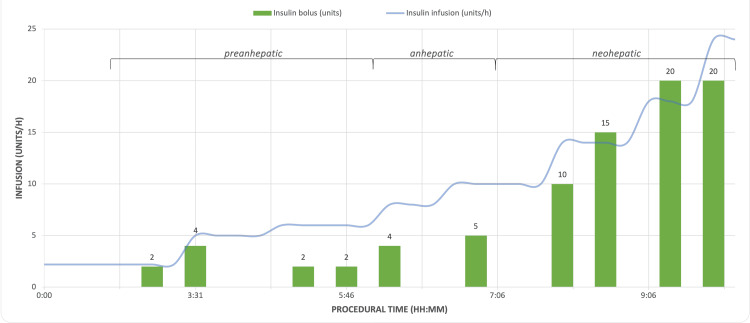
Intraoperative insulin requirements during domino liver transplantation Image Credit: Colton Southard-Goebel

The patient experienced gradual refractory intraoperative hyperglycemia, peaking in the neo-hepatic phase, requiring frequent insulin boluses, and a progressive increase in the basal rate up to 24 units/hour at the end of surgery (Figure 1). His intraoperative insulin requirements over 10.5 hours totaled 177 units (Figure 2). The intraoperative blood loss was 300 ml and 250 ml of salvaged blood via cell saver was returned to the patient. No additional blood products were required. Intraoperative fluid therapy included PlasmaLyte, totaling 7662 ml, and 500 ml of 5% albumin. In addition, dextrose 10% in sodium chloride (NaCl) 0.9%, which was quickly discontinued due to glycemic lability. There was moderate hemodynamic instability entering the anhepatic stage and vena cava cross-clamp. Norepinephrine, epinephrine, and vasopressin were titrated accordingly.

The patient remained intubated and was taken directly to the ICU at the end of the case. He was hemodynamically stable and did not require vasopressor support. Postoperative pain was well controlled with patient-controlled analgesia (PCA), methocarbamol, and acetaminophen. Alprostadil was initiated on postoperative day (POD) 0 due to concern for hepatic hypoperfusion. A repeat right-upper-quadrant ultrasound and liver enzymes suggested improving hepatic function and alprostadil was discontinued. The patient was extubated on POD 1 and transitioned to a nasal cannula. Carvedilol 6.25 mg was started on POD 5, and up titrated to 25 mg twice daily. He received several infusions of albumin during his hospitalization. His bilirubin rose again on POD 5 without concerning findings on repeat liver Doppler. The bilirubin subsequently downtrended and normalized on discharge. Ursodiol was added on POD 7.

TPN was discontinued on POD 6, EndoTool was discontinued on POD 7, and the home insulin pump resumed as glucose stabilized. Stable, uneventful postoperative hospital course with steadily down trending liver function tests (LFTs), transfer out of SICU on POD 8, and discharge home on POD 11.

As LFTs began to downtrend, the patient was initiated on steadily increasing intact protein diet per pediatric metabolism recommendations (0.8-1.0 g/kg/d). The patient could now consume a complete, normal, carb-consistent diet with no protein restriction for the first time in his life.

His aspartate aminotransferase (AST)/alanine transaminase (ALT) continued to improve significantly with no signs of acute transplant rejection. The patient was in high spirits, grateful, and eager to receive a pancreatic transplant in the future. At the follow-up three days post-discharge, blood glucose control was excellent and stable. At the preoperative baseline, the patient was determined to be at high risk for cytomegalovirus (CMV) due to a positive donor test and a negative recipient test. He was initiated on standard prophylaxis and testing. At the follow-up one month post-transplant, the incision was healing well, staples were removed, recovery was good after a norovirus infection, and blood glucose control remained excellent. At the follow-up four months post-transplant, the patient presented with elevated liver enzymes. Core needle biopsy demonstrated cellular changes consistent with mild to moderate cellular rejection with a Banff rejection activity index (RAI) of 5-6 out of 9. CMV and Epstein-Barr virus (EBV) polymerase chain reaction (PCR) were negative. The patient was admitted to the ICU and discharged four days later for outpatient management with pulse steroids and an oral steroid taper. His liver biochemistry quickly returned to baseline.

## Discussion

The current report described the case of a young adult male with comorbid MSUD and TIDM complicated by worsening encephalopathic crises, who presented for a DLT from a brain-dead donor. Following preoperative metabolic optimization, the patient experienced marked intraoperative hemodynamic and glycemic lability entering the anhepatic and neohepatic phase, respectively. The patient remained intubated at case conclusion with an otherwise unremarkable hospital stay, initiation of a non-protein-restricted diet, and prompt discharge. One month after the transplant, the patient showed complete resolution of MSUD symptoms, excellent blood glucose control, and significantly improved quality of life while consuming a regular diet.

MSUD is an inherited metabolic disease resulting from *BCKAD* dysfunction, which leads to impaired BCAA metabolism, accumulation of toxic metabolites, and consequently neurological damage. In 1964 the United States implemented routine universal newborn screening for MSUD. Management of MSUD entails metabolic monitoring and dietary BCAA restriction, positive clinical outcomes can be expected with prompt, fastidious management [2]. Notwithstanding efforts to manage MSUD through non-surgical approaches and strict adherence to metabolic requirements, encephalopathic crises are practically unavoidable [3].

The associated costs of this restrictive lifestyle to MSUD patients, their families, and the healthcare system are substantial [2,9]. DLT has been established as an effective, curative treatment for MSUD patients, providing critical benefits to the MSUD graft donor and recipient, simultaneously increasing the liver donor pool [2,5,6]. MSUD has an estimated worldwide incidence of one in 185,000 live births with a higher incidence in certain demographics, such as the Mennonite community (1 in 380 newborns) [1]. T1DM is an autoimmune disease characterized by an inability to produce insulin, with an estimated worldwide incidence of one in 300 [10]. Currently, there is one case report describing the co-occurrence of these two diseases [7]. Anecdotally, the patient in the current case mentioned encountering another individual with MSUD and T1DM from Israel through an international support group.

Brittle (or labile) diabetes is used to describe patients with frequent, severe disruptions in glycemia, occurring in roughly three out of every 1000 patients with insulin-dependent diabetes. Rarely, brittle diabetes can be secondary to an underlying medical condition, such as MSUD, causing disruption in insulin sensitivity or glucose utilization [11]. One theory regarding BCAA metabolism suggests a harmful buildup of byproducts, rather than the BCAAs themselves, leading to mitochondrial dysfunction, stress signaling, and apoptosis in pancreatic beta cells worsening insulin resistance. Another dysmetabolic theory purports that insulin resistance may contribute to aminoacidemia by increasing the protein catabolism, which insulin typically inhibits, and/or by impairing tissue-dependent BCAA oxidative metabolism. Patients with comorbid MSUD and T1DM are at elevated risk for DKA due to both diseases being ketotic processes, and both likely contributing to insulin resistance [5]. Our patient experienced increasingly frequent hospitalizations for DKA with and without leucine encephalopathy. Therapeutic and prophylactic management of hyperleucinemia frequently necessitates administering large doses of glucose to mitigate catabolic processes [1]. Nevertheless, in this patient with insulin-dependent diabetes, achieving an optimal balance poses a considerable challenge.

**Figure 3:**
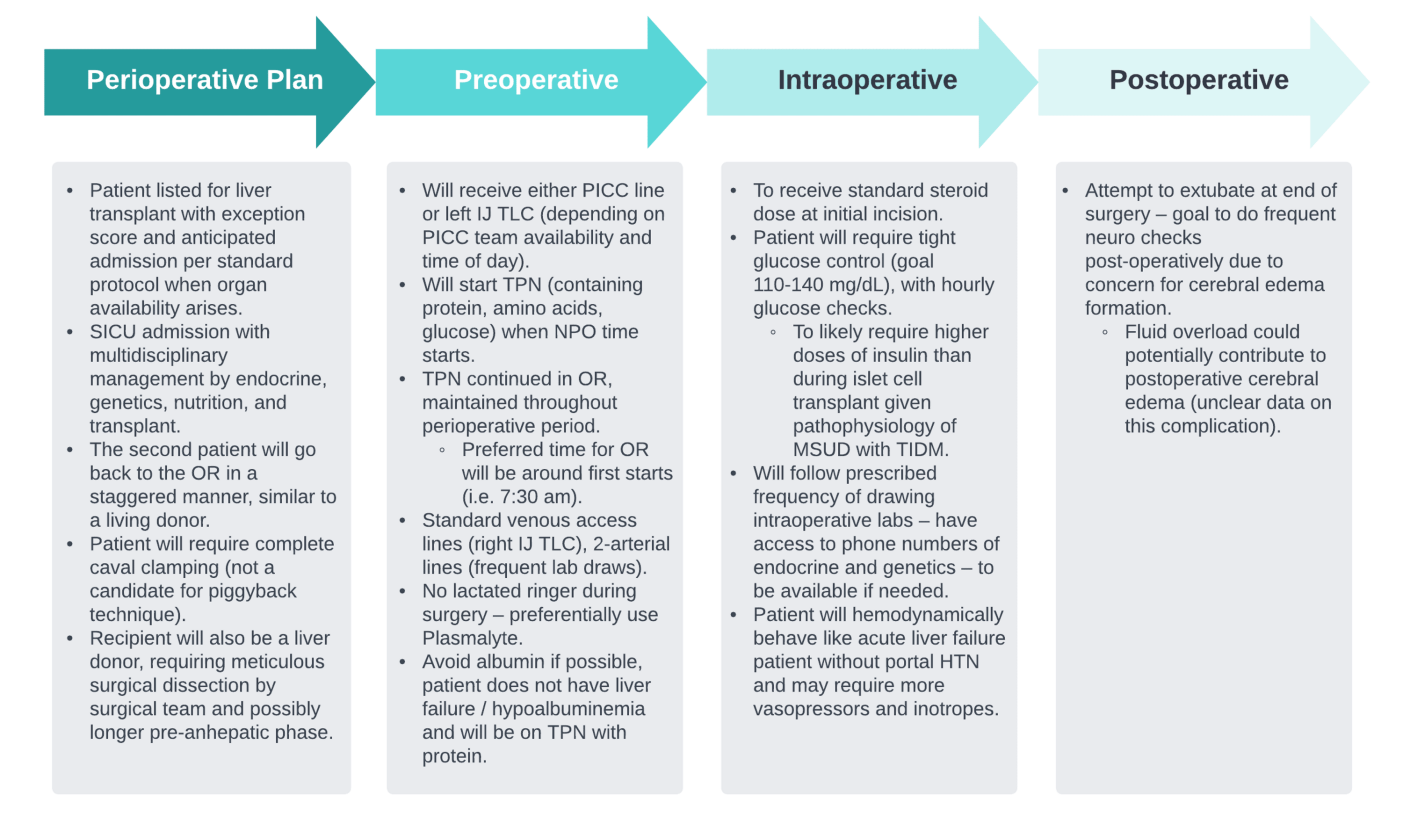
Summary of perioperative considerations PICC: peripherally inserted central catheter; IJ: internal jugular vein; TLC: triple lumen catheter Image Credit: Colton Southard-Goebel

In addition to the patient’s intrinsic diabetogenic traits, extrinsic factors such as corticosteroids, surgical stress, blood transfusions, and vasoactive drugs exacerbate intraoperative hyperglycemia [12,13]. Persistent neohepatic hyperglycemia as well as temporal, intraoperative glucose variability may serve as prognostic factors for early graft dysfunction. Glucose influx from the grafted liver, which is accelerated during rewarming and reperfusion, is the predominant contributor to neohepatic hyperglycemia. Due to insufficient clinical data, no standard protocol for glucose management during liver transplant exists [12]. In the perioperative period, literature suggests minimizing pre-surgical fasting with close monitoring of leucine, pH, glucose, fluid, and electrolytes, as insulin can redistribute, to diminish catabolic stress response with resultant hyperleucinemia and hyperglycemia [2]. A non-exhaustive list of perioperative considerations is summarized in Figure 3.

There was an emphasis on preoperative metabolic optimization for the current patient with well-documented, uniquely elevated baseline insulin requirements for his body habitus at 6.5 units/hour versus a standard 1-2 units/hour. Strict glycemic control parameters were initiated via EndoTool, with a goal range of 110-140 mg/dL. Intensive insulin therapy may decrease the rate of liver graft rejection in diabetic patients [14]. Retrospectively, intraoperative hyperglycemia during liver transplantation was associated with significantly increased post-transplant infection rate, one-year mortality, and one-year graft rejection [13]. Another large retrospective study described almost double the risk of reoperation for hemorrhage with higher intraoperative glycemic variability (SD ≥ 55 mg/dL) [12]. However, massive intraoperative blood loss results in glycemic instability and is itself the main determinant of surgical re-intervention in liver transplants. Nonetheless, in vitro studies demonstrate abrupt glycemic derangements can adversely affect coagulation and fibrinolysis [13]. No prospective studies exist examining the relationship between intraoperative hyperglycemia in liver transplants and post-transplant outcomes [12].

Our patient underwent 14 intraoperative blood glucose measurements, from arterial blood gas draws, with a mean blood glucose concentration of 182 mg/dL. Ammori et al. examined intraoperative hyperglycemia in orthotopic liver transplantation (OLT) and reported a total average insulin requirement of 24 ± 2 units and 13 ± 3 units in 184 patients undergoing OLT with poor glycemic control (mean blood glucose >150 mg/dL) and strict glycemic control, respectively [13]. Using Ammori et al.’s methodology, our patient would be defined as having poor intraoperative glycemic control (mean blood glucose >150 mg/dL) and falling neatly within the average blood glucose range of the poor glycemic control group (183 ± 2 mg/dL). During the 10.5-hour surgery, our patient required 177 units of insulin. Considering this drastic difference, it is crucial to note that Ammori et al.’s sample included patients with and without pre-existing diabetes, none of whom underwent DLT, a characteristically time-consuming hepatectomy [6,13].

Poor relative tolerance of caval cross-clamp and subsequent hemodynamic compromise is likely secondary to lack of vascular collateralization in an otherwise morphologically, healthy liver.

Liver volumetry is essential to optimizing donor and recipient outcomes in living donor liver transplantation [15]. On review of the electronic medical record (EMR), it is unclear as to whether liver volumetry was employed with our patient.

Acute cellular rejection (ACR) occurs in 10-30% of liver transplant recipients. Unlike other solid organs, liver ACR rarely threatens graft survival and usually responds to steroids. The etiology of our patient’s ACR is unknown and likely multifaceted, potentially involving autoimmune or infectious factors, immunosuppressive noncompliance, or intraoperative variables such as prolonged cold-ischemia time [16].

Since the first case in 2005, a total of 54 patients worldwide have been reported to have received the liver of an MSUD patient [5]. There have been no reported cases of novel MSUD development in the graft recipient, no difference in postoperative recovery metrics compared with the MSUD patient, and primary disease relief in all MSUD patients [5,17]. The majority of MSUD DLT allocation does not follow the classical liver distribution system, with many scholars arguing for uniform distribution of this valuable resource [5,6].

## Conclusions

The clinical presentation of MSUD and TIDM in an adult has not been previously reported. The comorbidity of labile TIDM is a complicating factor in DLT, but not a prohibitive one. This report supports the existing literature that MSUD DLT is safe, efficacious, and sustainable, sparing net loss of graft from the limited donor pool. We support the call for further standardization of MSUD liver graft allocation to provide equitable access to this life-saving resource. Further research is warranted to better understand the long-term prognosis of DLT and quality of life in patients with comorbid MSUD and TIDM, given the complex interplay between these two pathologies.
